# Resuscitative endovascular balloon occlusion of the aorta (REBOA) during cardiac resuscitation increased cerebral perfusion to occurrence of cardiopulmonary resuscitation-induced consciousness, a case report

**DOI:** 10.1016/j.resplu.2024.100646

**Published:** 2024-04-27

**Authors:** Jostein Rødseth Brede, Eivinn Årdal Skjærseth

**Affiliations:** a Department of Emergency Medicine and Pre-Hospital Services, St. Olav University Hospital, Trondheim, Norway; b Norwegian Air Ambulance Foundation, Department of Research and Development, Oslo, Norway; c Department of Anaesthesiology and Intensive Care Medicine, St. Olav́s University Hospital, Trondheim, Norway

**Keywords:** CPR, CPRIC, Resuscitation consciousness, OHCA, Cardiac arrest, REBOA, Aortic occlusion

## Abstract

Consciousness or signs of life may be seen during cardiopulmonary resuscitation (CPR), without return of spontaneous circulation. Such CPR-induced consciousness includes breathing efforts, eye opening, movements of extremities or communication with the rescuers. The consciousness may be CPR-interfering or non-interfering, and typically ends when the resuscitation efforts end. Resuscitative endovascular balloon occlusion of the aorta (REBOA) is a potential adjunct treatment to CPR and may increase the arterial blood pressure. We present a case where REBOA increased the arterial blood pressure to the extent that CPR-induced consciousness was seen.

## Introduction

**Fig. 1:**
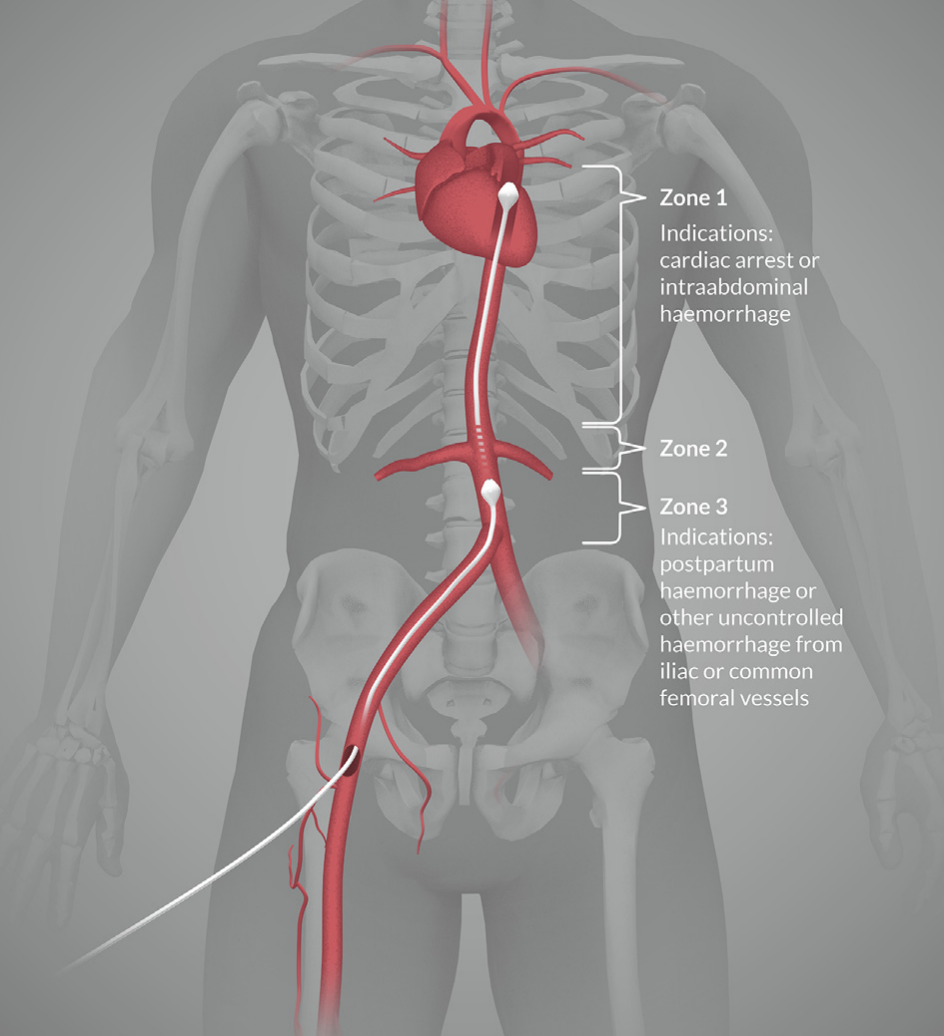
Aortic zones and indications for resuscitative endovascular balloon occlusion of the aorta.^6^

Cardiopulmonary resuscitation (CPR) aims to improve oxygen delivery to the brain and the heart during cardiac arrest, to prevent or delay hypoxic damage to the brain and achieve return of spontaneous circulation (ROSC).^1^ Resuscitative endovascular balloon occlusion of the aorta (REBOA) is a well-known technique to limit blood loss in management of major trauma^2^ (Fig. 1). However, REBOA may also be an adjunct treatment in non-traumatic cardiac arrest, due to a potential increase in aortic blood pressure.^3^, ^4^ We present a case where REBOA increased the blood pressure sufficient to generate CPR-induced consciousness (CPRIC).

This case report is reported according to the CARE guidelines^5^ and written informed consent has been provided by the patient’s next-of-kin.

## Case description

A previously healthy 71-year-old male sustained an out-of-hospital cardiac arrest (OHCA) at his home. Both the nearest ground ambulance and the helicopter emergency medical service (HEMS) were dispatched to the scene. Norwegian HEMS includes a consultant anesthesiologist and a paramedic.^7^ The ground ambulance arrived 14 min after dispatch and suspected a non-traumatic etiology of the arrest. The initial cardiac rhythm was a pulseless electrical activity (PEA) and the ambulance crew started resuscitation according to the national guidelines.^8^ A chest compression machine (LUCAS^TM^ CPR, Physio Control-Inc, Lund, Sweden) was used.

HEMS arrived 30 min after dispatch and the cardiac rhythm was still PEA. The patient was endotracheally intubated and an arterial cannula for invasive blood pressure measurements was placed in the left radial artery.

The HEMS crew were at that time involved in a cardiac arrest trial were REBOA was used as an adjunct to CPR.^9^, ^10^ The crew had participated in structural REBOA training and performed the procedure on selected patients according to a standard operating procedure.^11^

The patient was included to the ongoing trial and 11 min after HEMS arrival the crew started the REBOA procedure. Eight minutes were used from start of procedure to balloon inflation, hence aortic occlusion was performed 49 min after HEMS dispatch. The REBOA procedure was registered as uneventful in the journal, with easy visualization of the femoral artery and vein with ultrasound, easy percutaneously arterial cannulation, insertion of guidewire, introducer, and the 7 French balloon sheath (Reboa Balloon Kit, 7 Fr, 20 mm, Reboa Medical AS, Norway). No interruption of the ongoing CPR was registered.

**Fig. 2:**
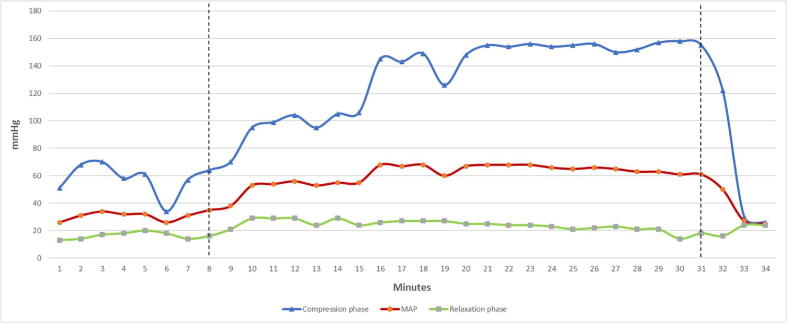
Blood pressure measurements during resuscitation. The dotted line at 8 min indicates balloon inflation, the dotted line at 31 min indicates balloon deflation. MAP indicates mean arterial pressure.

The end-tidal CO2 value prior to balloon inflation was 3.4 kPa, which increased to 4.4 kPa immediately after occlusion, 4.0 after 30 s and 4.0 after 90 s. No further EtCO2-values were registered in the journal. The blood pressure increased after aortic occlusion, as shown in Fig. 2.

Shortly after aortic occlusion, the patient demonstrated signs of CPRIC, with normalized breathing efforts at 12–15 breaths per minute and eye opening, without ROSC. The consciousness did not interfere with the resuscitation and the physician did not administer additional pharmacological treatment during CPR.

Due to signs of life during CPR, the HEMS crew contacted the on-call thoracic surgeon at the referral university hospital to consult if the patient was a candidate for extracorporeal membrane oxygenation treatment. This was declined due to patient age, initial rhythm, and prolonged cardiac arrest, and signs of life were not regarded as sufficient indication. The patient had now been in cardiac arrest for more than 70 min without any episodes of ROSC, and the health care providers at the scene decided to stop the resuscitation efforts. The balloon was deflated, and the blood pressure dropped immediately (Fig. 2). Two minutes after deflation the chest compressions were stopped, and the patient was declared dead.

## Discussion

The CPRIC phenomenon was first described in 1989.^12^ CPRIC may be seen when the cerebral circulation is sufficient to restore some of the cerebral functions, however without return of spontaneous circulation (ROSC).^1^, ^13^ It includes signs of life such as breathing efforts, eye opening, movements of extremities or communication with the rescuers,^13^ and may be classified as CPR-interfering or non-interfering.^13^, ^14^ The consciousness ends when the resuscitation ends. The prevalence of CPRIC is reported to 0.23–0.9% of cardiac arrests, however these numbers are unreliable due to the design of the studies.^15^ The true prevalence of the phenomenon is therefore uncertain. In Norway there is a nation-wide increased use of chest compression machines, increasing from 28% in 2015 to 33% in 2021.^16^ Use of chest compression machines may facilitate high quality chest compressions without interruptions and can therefore increase the prevalence of CPRIC. Earlier findings suggest that CPRIC has been observed by 48–59% of prehospital health care providers.^15^, ^17^ However, a recent national survey amongst prehospital anesthesiologist in Norway report that 91% of the physicians had observed CPRIC,^14^ and a chest compression machine was used in 79% of the cases. This may suggest both that CPRIC is more common than previously believed, and that the use of chest compression machines may contribute to this. One example is a recent case report where the resuscitation of a 17-year-old were successful after use of REBOA and left stellate ganglion block.^18^ CPRIC was seen after initiation of a chest compression device, prior to the use of REBOA.

It has been demonstrated that REBOA as an adjunct to CPR may increase the peripheral blood pressure^10^, ^19^ and the aortic root blood pressure.^3^ This have led to the suggestion that REBOA may be utilized in non-traumatic cardiac arrest patients.^20^ The use of REBOA as an adjunct treatment is currently investigated in a randomized controlled clinical trial.^21^ Notably, it was the compression phase pressure that increased in this case (Fig. 2). This may contribute to increased cerebral perfusion and the CPRIC phenomenon. However, CPRIC is not likely caused by increased blood pressure alone. Factors such as the patients’ autoregulation, ischemic threshold, and comorbidities all influence brain oxygenation, hence the chance of generating sufficient cerebral perfusion to achieve resuscitation consciousness.

The relaxation phase pressure, however, did not increase. It is demonstrated that the coronary perfusion pressure during the relaxation phase is related to ROSC in humans,^22^ which may explain why ROSC was never achieved in this patient.

It is unknown if and how CPRIC should be managed, or if CPRIC is a positive prognostic factor. Some prehospital treatment protocols are published,^23^ but there is a lack of international or national consensus on intra-arrest management. The 2021 consensus statement from the International Liaison Committee on Resuscitation included a good practice statement where small doses of sedative and/or analgetic drugs could be considered. No specific drug regimen was recommended, however the use of muscle relaxant alone was not recommended.^24^ In a recent nationwide survey amongst prehospital anesthesiologist in Norway, 50% suggested that sedation should be provided to patients with CPRIC. Twenty percent answered that no sedation was needed, while 30% was uncertain. Fentanyl, ketamine, and midazolam was reported as the most preferred sedation agents. Interestingly, physical restraint was not recommended by any of the physicians, however it was reported used in 23% of the cases. Further, muscle relaxant was recommended by 14% of the physicians, but reported used by 27%. This may indicate that CPRIC is often CPR-interfering and that a consensus for management of these patients is warranted, as experienced prehospital physicians have considerable variability in treatment of these patients.

## Conclusion

We present a case where REBOA used during CPR contributed to an increase in arterial blood pressure to the extent that CPR-interfering resuscitation consciousness was seen. The case highlights the potential hemodynamic effect of aortic occlusion during CPR, and the need to assess how resuscitation consciousness may impact patient treatment and outcome.

## Ethics

The patient was included in a study approved by the Norwegian Regional Committee for Medical and Health Research Ethics (reference 2018/51/REKMidt) and registered at ClinicalTrials.gov (Identifier: NCT03534011). The patients’ next-of-kin provided written informed consent for the use of the data and to publish this case report.

## Funding

The funding for open access is granted by the Norwegian Air Ambulance Foundation, Oslo, Norway. The authors received no external funding.

## Availability of data and materials

The dataset used may be made available from the corresponding author upon reasonable request.

## CRediT authorship contribution statement

**Jostein Rødseth Brede:** Writing – review & editing, Writing – original draft, Visualization, Data curation, Conceptualization. **Eivinn Årdal Skjærseth:** Writing – review & editing, Data curation, Conceptualization.

## Declaration of competing interest

The authors declare the following financial interests/personal relationships which may be considered as potential competing interests: ‘Dr Jostein Rødseth Brede is partly employed for research purposes in the Norwegian Air Ambulance Foundation. Dr Eivinn Skjærseth declares no conflict of interest’.
